# Potential Benefits and Risks Associated with the Use of Statins

**DOI:** 10.3390/pharmaceutics16020214

**Published:** 2024-02-01

**Authors:** Nisha Khatiwada, Zhongkui Hong

**Affiliations:** Department of Mechanical Engineering, Texas Tech University, Lubbock, TX 79409, USA; nikhatiw@ttu.edu

**Keywords:** statin, pleiotropic effects, statin-associated symptoms

## Abstract

HMG-CoA reductase inhibitors, commonly known as statins, are the primary treatment choice for cardiovascular diseases, which stand as the leading global cause of mortality. Statins also offer various pleiotropic effects, including improved endothelial function, anti-inflammatory properties, reduced oxidative stress, anti-thrombotic effects, and the stabilization of atherosclerotic plaques. However, the usage of statins can be accompanied by a range of adverse effects, such as the development of type 2 diabetes mellitus, muscular symptoms, liver toxicity, kidney diseases, cataracts, hemorrhagic strokes, and psychiatric complications. These issues are referred to as statin-associated symptoms (SAS) and are relatively infrequent in clinical trials, making it challenging to attribute them to statin use definitively. Therefore, these symptoms can lead to significant problems, necessitating dose adjustments or discontinuation of statin therapy. This review aims to provide a comprehensive overview of the mechanism of action, potential advantages, and associated risks of statin utilization in clinical settings.

## 1. Introduction

According to statistics provided by the World Health Organization (WHO), cardiovascular disease (CVD) was the primary cause of death on a global scale in 2019, accounting for 32% of all fatalities [1]. Data collected from 2015 to 2018 indicates that 38.1% of adults in the United States, equivalent to 93.9 million individuals, had total cholesterol levels equal to or exceeding 200 mg/dL [2]. Furthermore, elevated levels of low-density lipoprotein cholesterol were responsible for 4.51 million deaths worldwide in 2020, indicating a 19% increase compared with 2010 [3]. Increased levels of low-density lipoprotein (LDL) lead to a condition called atherosclerosis, where excessive cholesterol accumulates in various arteries throughout the body. The buildup of LDL in the coronary vessels and carotid arteries increases the risk of experiencing a myocardial infarction or stroke [4,5,6].

Atherosclerosis is no longer limited to Western countries. It has become a global health concern, affecting younger individuals, a wider range of ethnic backgrounds, and more women [7]. Statins have served as the primary treatment for preventing CVDs for many years, starting with the introduction of lovastatin in 1987, which was the first statin available for commercial use [8]. These medications work by competitively inhibiting HMG-CoA reductase, a crucial enzyme that controls the rate of cholesterol production in the liver. This inhibition leads to a decrease in cholesterol synthesis within the liver. In response to the lowered cholesterol, hepatocytes increase the production of LDL receptors, enhancing the uptake and recycling of LDL cholesterol. Consequently, this process results in reduced levels of LDL cholesterol in the bloodstream. This reduction in serum LDL levels is the primary mechanism through which statin therapy decreases the risk of cardiovascular problems and improves overall outcomes [9,10]. One early study observed a dose-dependent decrease in LDL cholesterol levels, ranging from 25% to 60%, with atorvastatin therapy [11]. A number of research studies have investigated the role of statin therapy in lowering the occurrence of significant vascular events [12,13]. These studies consistently demonstrate a strong correlation between the extent of reduction in LDL cholesterol levels among patients and the reduction in cardiovascular events.

Statins can be categorized into different classes based on either their origin or chemical structure. Regarding their origin, there are three classes: natural, semi-synthetic, and synthetic. Natural statins are derived from the fermentation of fungi and encompass lovastatin and pravastatin [14]. Semi-synthetic statins, such as simvastatin, are produced through a process that involves modifying lovastatin, specifically by substituting the 2-methylbutyrate group at the C-8 position of the naphthalene ring with 2,2-dimethylbutyrate [15]. Synthetic statins are chemically synthesized and share only the common dihydroxyheptanoic acid pharmacophore (resembling HMG-CoA) that mimics the HMG-CoA substrate in binding to HMGR [16]. Examples of synthetic statins include atorvastatin, cerivastatin, rosuvastatin, fluvastatin, and pitavastatin. All forms of statins can undergo reversible metabolism through lactonization [17].

In general, statins are considered safe and well-tolerated medications, and they are the most commonly prescribed drugs worldwide. Clinical trials have shown that statins effectively lower LDL cholesterol levels and improve patient prognosis without increasing complications [18]. These drugs also have additional benefits, such as reducing inflammation markers such as C-reactive protein and pro-inflammatory cytokines. Statins improve endothelial function in patients with cardiovascular risk factors and are associated with protective effects in coronary artery disease (CAD) [12,19]. They also play a role in preventing stroke, improving outcomes in acute coronary syndrome, reducing the risk of atrial fibrillation after heart surgery, and benefiting patients with heart failure. Statin treatment has demonstrated cardiovascular risk reduction, even in healthy individuals with elevated inflammation markers [20,21]. Additionally, the regulation of hepatic LDL-receptor-related protein 1 expression is considered a mechanism underlying the protective effects of statins on the cardiovascular system [22,23]. According to a study conducted from 2002 to 2013 using Medical Expenditure Panel Survey data, the use of statins among adults aged 40 and older in the US increased by 79.8% from 21.8 million people (17.9%) in 2002–2003 to 39.2 million people (27.8%) in 2012–2013 [24].

In addition to their positive impact on vascular health, many studies have highlighted the potential pleiotropic effects of statins that could affect various tissues and organs. Although most patients can address adverse effects by trying a different statin or adjusting the treatment plan, such as reducing the dosage or combining statins with non-statin drugs, current guidelines favor the term “statin-associated side effects” over “statin intolerance”. Evaluating and addressing these adverse effects can be challenging, even though they occur infrequently in clinical studies. The most common of these are statin-associated muscle symptoms (SAMS), typically characterized by subjective muscle pain and are observed in approximately 5% to 20% of patients. In some susceptible individuals, statins slightly elevated the risk of developing diabetes mellitus [25]. The risk of significant liver damage induced by statins is extremely low, estimated at around 0.001%. Additionally, statins may increase the risk of hemorrhagic stroke in individuals with cerebrovascular disease [26].

Some individuals fail to achieve the desired LDL-C target levels or encounter challenges in tolerating statins, especially when on high doses for extended periods. The future focus on reducing the burden of CVD involves combining statins with diverse therapeutic approaches and exploring new drugs [27]. In recent times, there has been significant progress in the development and approval of various cholesterol-lowering medications, expanding the range of pharmacological options beyond statins. Emerging agents such as PCSK9 inhibitors, bempedoic acid, inclisiran, ANGPTL3 inhibitors, PPARβ/δ agonists, and LXR agonists show promise in effectively reducing LDL-C levels, demonstrating encouraging positive outcomes [28].

In this review, we provide an overview of recent research advancements, highlighting both the positive and negative effects of statin therapy on human health. It stresses the importance of considering these factors before initiating statin therapy and underscores the crucial role of understanding side effects and their underlying mechanisms for early detection, prevention, and management. The comprehensive exploration of statin therapy includes a patient-centered approach, precision medicine perspective, and consideration of combination therapies. Prioritizing a patient-centered approach involves understanding preferences, concerns, and experiences, while precision medicine in statin prescriptions explores genetic factors and individualized patient data. This review also emphasizes evaluating the long-term impact of statin therapy on aging populations, assessing combined interventions for cardiovascular risk reduction, and examining statins’ effects beyond cholesterol reduction. Addressing adverse effects incorporates lifestyle modifications. The integration of real-world evidence and health economics assesses statin therapy’s cost-effectiveness, considering global variations and health disparities in use, accessibility, and outcomes. Overall, this review advocates for a nuanced and inclusive approach to statin therapy, considering its multifaceted aspects for better patient outcomes.

## 2. Pleiotropic Benefits of Statin

**Figure 1:**
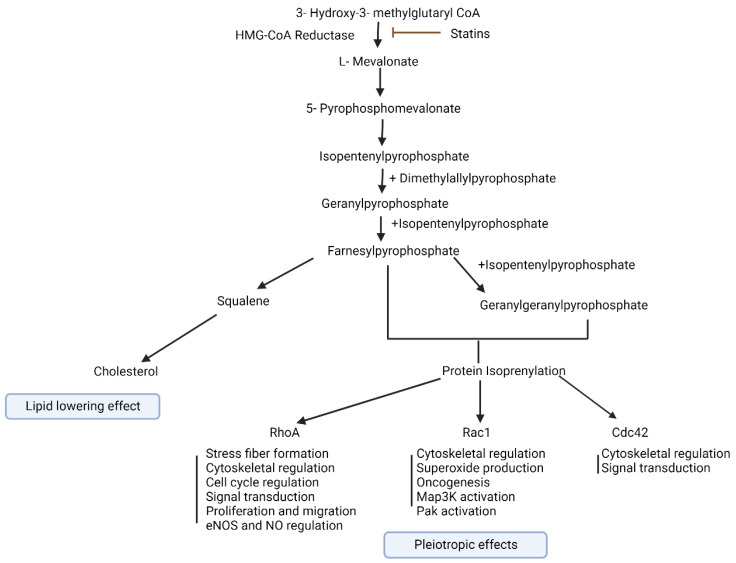
Cholesterol and isoprenoid synthesis pathway illustrating how statins inhibit the enzyme 3-hydroxy-3-methyl-glutaryl-coenzyme A (HMG-CoA) reductase. Created with BioRender.com. Accessed on 4 October 2023.

Statins lower cholesterol synthesis in the liver by reducing the production of mevalonate [29]. Figure 1 represents the pathway of cholesterol and isoprenoid synthesis demonstrating how statins impede the activity of the enzyme 3-hydroxy-3-methyl-glutaryl-coenzyme A (HMG-CoA) reductase. This mechanism helps reduce the risk of atherosclerosis by decreasing the likelihood of cholesterol accumulation. Lower cellular cholesterol levels encourage the presentation of LDL receptors on the surface of liver cells (hepatocytes), prompting the clearance of LDL cholesterol from the bloodstream [9]. Additionally, statins lead to higher levels of beneficial high-density lipoprotein (HDL) cholesterol while reducing serum triglycerides [10]. They primarily affect the internal lipid metabolism pathway of the body rather than the external one [13]. Furthermore, statins are associated with benefits such as stabilizing arterial plaques, preventing blood clot formation, and enhancing the health of blood vessel endothelium.

According to the results of a meta-analysis, the decreases in significant coronary events [30,31], coronary revascularizations, and strokes were associated with the actual reductions in LDL cholesterol achieved through the examined statin regimens [19]. Statins have been demonstrated to enhance endothelial function, smooth muscle cell function, and the functioning of monocyte-macrophage cells in clinical scenarios. These positive effects may not be solely attributed to lipid-related processes but also involve improvements in vasomotor function, the regulation of inflammatory responses, and the stability of arterial plaques [32].

**Figure 2:**
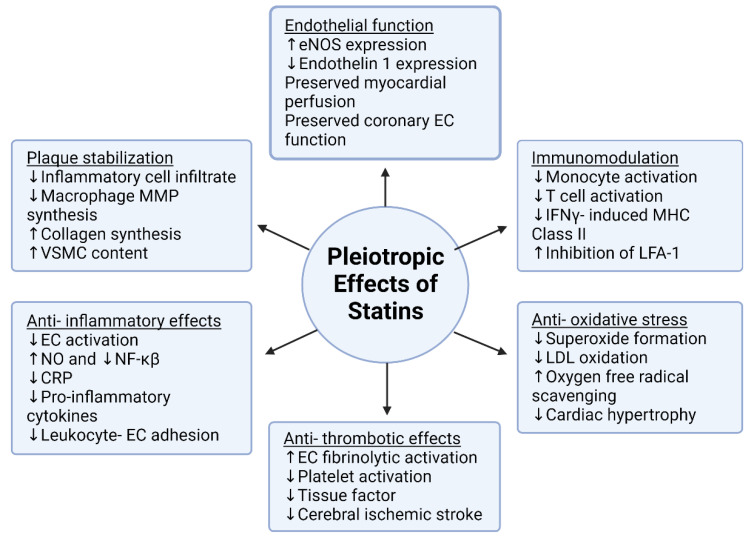
Pleiotropic Benefits of Statins. Created with BioRender.com. Accessed on 6 October 2023.

Research involving animals and experiments has shown that statins can lower plasma cholesterol levels without impacting the migration or proliferation of smooth muscle cells [33]. Additionally, statins can reduce the production of matrix metalloproteinases and the in vitro accumulation of cholesterol in macrophages, thereby promoting plaque stability. Statins have been observed to decrease the synthesis of pro-inflammatory cytokines, C-reactive protein, cellular adhesion molecules, and the activation of monocytes into macrophages. Furthermore, statins reduce the adherence of monocytes to endothelial cells [34]. Consequently, apart from their effects on blood lipid levels, statins exert direct cardiovascular benefits through their antiatherogenic properties within the artery wall [32]. A recent study proposed that statins slow down the progression of atherosclerotic disease in patients by reducing the cholesterol content within vascular smooth muscle cells (VSMCs) and aortic tissue and by modifying their biomechanical function [35]. Figure 2 shows a comprehensive depiction of the pleiotropic benefits attributed to statins. It illustrates the involvement of statins in diverse physiological processes, modulating various cellular functions through activation and deactivation. In the Figure 2, upward arrows signify an increase, while downward arrows indicate a decrease.

### 2.1. Enhancing Endothelial Function

The onset of atherosclerosis often involves endothelial dysfunction, which is influenced by established cardiovascular risk factors such as hypertension, smoking, and high blood sugar levels. These factors, mediated by nitric oxide (NO), can interfere with the regular vasodilation process. Statins play a role in inhibiting the prenylation of Rac and Rho proteins, leading to an increase in the production of endothelium-derived nitric oxide synthetase (eNOS). This, in turn, enhances the production of nitric oxide and supports vasodilation [36]. This increased eNOS expression results in the elevated production of nitric oxide within the endothelium, which supports vasodilation [37,38]. NO release serves not only to facilitate vasodilation but also to hinder leukocyte adhesion, prevent platelet aggregation, and reduce the proliferation of vascular smooth muscle. As a result, NO plays a protective role, and inadequate levels indicate a greater risk of cardiovascular events [39].

**Figure 3:**
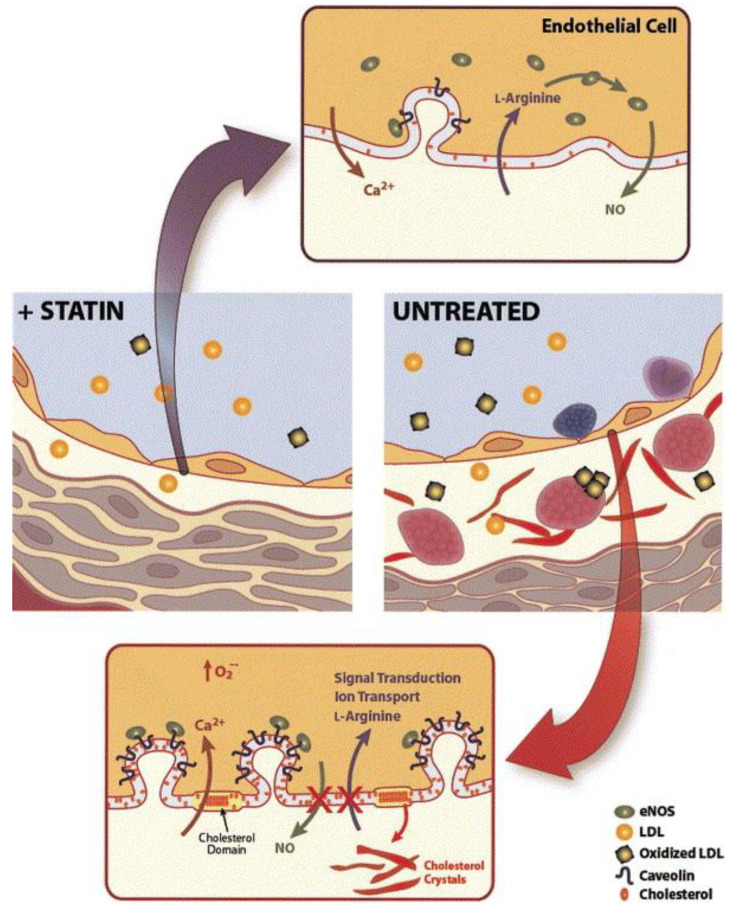
Alteration occurs in the vessel wall and the endothelial cell membrane during the development of atherosclerosis [40].

A schematic representation in Figure 3 shows alterations in the vessel wall and endothelial cell membrane that occur during hyperlipidemia. These changes include LDL introduction, monocyte adhesion, platelet aggregation, foam cell formation, and the accumulation of cholesterol crystals in the intima. Alterations in the endothelial cell membrane include the presence of cholesterol-rich domains and increased caveolae, where caveolin plays a key role in regulating eNOS. These modifications are attenuated by HMG-CoA reductase inhibitors, which lower LDL levels and inhibit cholesterol and isoprenoid biosynthesis pathways [38].

### 2.2. Plaque Stabilization

**Figure 4:**
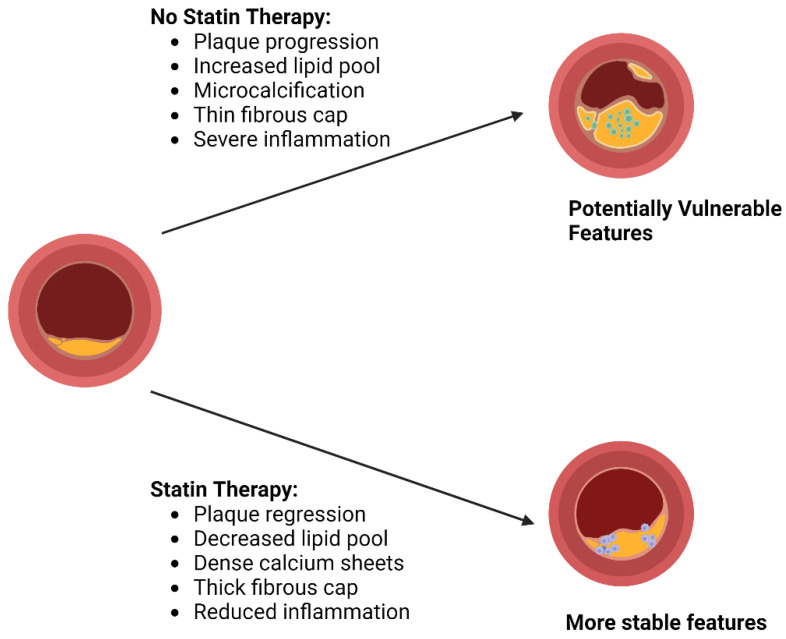
Statin therapy alters coronary plaque features. Created with Biorender.com. Accessed on 29 October 2023.

High-intensity statin therapy has been shown in various clinical trials to stabilize and even regress atherosclerotic plaque [41,42,43,44,45,46]. Trials such as REVEAL, ASTEROID, SATURN, and studies using advanced imaging techniques such as optical coherence tomography (OCT), coronary computed tomography angiography, and intravascular ultrasound (IVUS) technology have provided evidence of plaque stabilization and reduced progression in individuals on statin therapy [41]. In trials such as the REVEAL study, individuals receiving high-dose statins such as atorvastatin and rosuvastatin witnessed either a lack of atheroma volume progression or a reversal in atheroma growth within a relatively brief timeframe [42]. In the ASTEROID trial, rosuvastatin significantly reduced atherosclerotic plaque in patients over 24 months [43]. This significant regression in plaque underscores the effectiveness of high-intensity statin therapy in reducing plaque accumulation. Similarly, the SATURN trial provided further evidence supporting the efficacy of high-intensity statin treatment [44]. When comparing two high-intensity statins, rosuvastatin, and atorvastatin, it became apparent that both groups displayed a similar degree of atheroma regression after two years of therapy, indicating the ability of high-intensity statins to stabilize atherosclerotic plaques. Furthermore, advanced imaging methods such as optical coherence tomography (OCT) and coronary computed tomography angiography have yielded valuable information about plaque attributes. For instance, the EASY-FIT investigation revealed that atorvastatin enhanced the thickness of the fibrous cap, a crucial factor in maintaining plaque stability [45]. The PARADIGM study, utilizing coronary computed tomography angiography, uncovered that individual under statin treatment exhibited a decelerated rate of atheroma volume progression, increased plaque calcification, and a reduction in high-risk plaque characteristics. This finding is of significant importance as it indicates that statins play a role in maintaining plaque stability, ultimately reducing the likelihood of plaque rupture and related cardiovascular incidents [46]. These results highlight the effectiveness of high-intensity statins in improving atherosclerotic plaque characteristics without increasing stenosis severity, as demonstrated in Figure 4.

### 2.3. Anti-Inflammatory Effects

**Figure 5:**
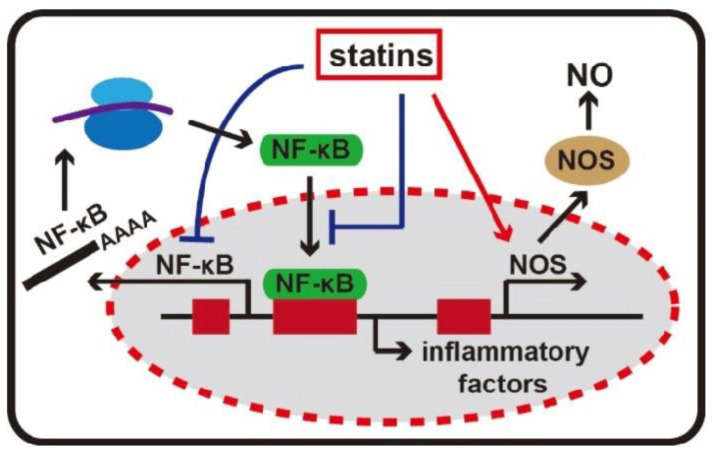
Anti-inflammatory effect of statins [47].

After endothelial damage, atherosclerotic plaques undergo infiltration of inflammatory cells. Statins can mitigate this inflammation by efficiently decreasing the production of inflammatory indicators, including C-reactive protein (CRP), serum amyloid A (SAA), interleukins, and adhesion molecules such as intracellular adhesion molecules (ICAM-I). These markers have all been associated with the initiation and recurrence of cardiovascular events. As illustrated in Figure 5, statins possess the ability to suppress NF-κB activity, a crucial transcription regulatory protein involved in inflammatory responses. Furthermore, statins can activate the transcription of the NOS gene, thereby enhancing NO production [38].

In one of the conducted cohort studies, pravastatin interestingly demonstrates a more substantial reduction in risk among individuals at a high risk of coronary events with elevated SAA and CRP levels compared with those with equivalent cardiovascular risk but normal levels of inflammatory markers [48]. This observation supports the notion that statins do not solely reduce risk by lowering cholesterol but also by suppressing inflammation. Importantly, both atorvastatin and simvastatin can reduce CRP levels, even in patients without elevated cholesterol [49,50], indicating that statins could be beneficial for individuals with normal LDL but heightened inflammatory markers.

### 2.4. Immunomodulatory Effects

There is informal evidence to suggest that statins may possess anti-inflammatory and immunomodulatory properties, which could be beneficial in conditions such as cardiac transplant rejection and various autoimmune diseases such as rheumatoid arthritis, ankylosing spondylitis, lupus, vasculitis, and systemic sclerosis [51]. Statins inhibit the induction of MHC-II expression by interferon γ (IFN-γ), leading to the repression of MHC-II-mediated T-cell activation. This effect is due to the inhibition of the inducible promoter IV of the transactivator CIITA and is observed in various cell types. MHC-II molecules play a crucial role in antigen presentation and T-cell activation through the T-cell receptor (TCR). TCR activation can impact T-cell proliferation, differentiation, and cytokine release. Cytokines released by activated T cells stimulate further T-cell proliferation, activate antigen-presenting cells (APCs), and promote B-cell antibody production. CD4+ helper T cells (TH cells) can differentiate into two distinct effector cell populations, TH1 and TH2, each producing different cytokines. Shifting the balance from TH1 to TH2 responses is beneficial in diseases characterized by delayed-type hypersensitivity reactions, such as graft atherosclerosis and chronic inflammatory conditions. Statins can induce this shift from TH1 to TH2 lymphocytes [52,53]. Both in vitro and in vivo studies suggest that statins may hinder the proliferation and cytotoxicity of T-lymphocytes and natural killer cells. Lastly, statins reduce the expression of cellular adhesion molecules found on both leukocytes and endothelial cells, leading to impaired cell adhesion and reduced migration to inflamed areas [54].

### 2.5. Anti-Thrombotic Effects

In the final stage of atherosclerosis, damage to the endothelium can lead to the formation of blood clots that obstruct blood flow. Statins can impede the formation of blood clots through multiple mechanisms, including a reduction in tissue factor expression and the inhibition of platelet aggregation [55,56,57]. This results in a decrease in thrombin production and the expression of its receptor on platelet surfaces. In addition to preventing blood clot formation, statins also promote the dissolution of clots by reducing levels of plasminogen activator inhibitor 1 (PA1-1) and enhancing the activity of the fibrinolytic enzyme plasminogen. The anticoagulant properties of statins were demonstrated in the JUPITER study, which showed a decreased rate of peripheral venous thromboembolism in patients taking rosuvastatin [58,59]. 

### 2.6. Reduced Oxidative Stress

Many studies suggest that statins have the potential to reduce oxidative stress through various mechanisms, including the inhibition of ROS production, enhancement of antioxidant defenses, protection of endothelial cells, and anti-inflammatory effects [60]. The metabolites of atorvastatin, particularly hydroxy metabolites, exhibit the ability to inhibit the oxidation of LDL, HDL, and VLDL particles. This suggests that statins may exert an antioxidant effect that could contribute to impeding the progression of atherosclerosis independently of their LDL-lowering effects [61]. Statins not only hinder the production of cholesterol but also disrupt the generation of Rac1, a protein involved in activating nicotinamide adenine dinucleotide phosphate (NADPH) oxidase and the production of reactive oxygen species (ROS) [62]. These ROS can contribute to various negative effects such as endothelial dysfunction, inflammation, and oxidation of LDL particles, all of which play roles in the development of atherosclerosis [63].

### 2.7. Protection from High-Decibel Noise-Inducing Hearing Loss

In a recent study, researchers evaluated the potential of statins as a treatment for hearing loss in CBA/CaJ mice [64]. They investigated the effects of delivering fluvastatin directly to the cochlea and administering lovastatin orally, assessing hearing outcomes using methods such as auditory brain stem responses (ABRs). Mice were exposed to two hours of octave band noise. Previous research with guinea pigs had demonstrated the protective effects of fluvastatin in the contralateral cochlea. Exposure to high-intensity noise (120 dB SPL for 4 h at 4–8 kHz) was found to result in the loss of hair cells—a phenomenon absent in guinea pigs subjected to noise but treated with fluvastatin. [65]. In the mouse study, hearing in the contralateral cochlea was evaluated 1–4 weeks after noise exposure. Two weeks post-exposure, mice treated with noise + carrier exhibited elevated ABR thresholds, whereas those treated with noise + fluvastatin showed smaller elevations. Oral lovastatin delivery resulted in lower threshold shifts compared with the carrier alone, indicating potential protection against noise-induced hearing loss (NIHL) [64]. These results suggest that statins may offer a protective effect against noise-induced damage to the auditory system, warranting further investigation into their potential benefits.

### 2.8. Enhance Responses to Immune Checkpoint Blockade in Cancer Models

In the early stages of statin research, concerns arose about a potential link between statin use and cancer risk, particularly with lipophilic statins such as simvastatin [66]. Early observational studies suggested a link between statins and an increased risk of certain cancers [67]. This concern originated from the understanding that statins, acting as cholesterol-lowering agents, influence cellular processes associated with cancer development. The reduction in cholesterol, crucial for cell membrane integrity, raised theoretical concerns about long-term consequences. However, as more robust studies unfolded, these early apprehensions lacked consistent support. Over time, a growing body of evidence has suggested that, in certain circumstances, statins may possess anti-cancer properties. 

A recently published study focused on the limited response of recurrent/metastatic head and neck squamous cell carcinoma (HNSCC) to first-line anti-PD-1 immune checkpoint blockade. Using murine models with oral cancer, the researchers found that all seven statins inhibited tumor cell proliferation, with simvastatin and lovastatin increasing T-cell killing. In mice, daily oral administration of simvastatin or lovastatin, combined with PD-1 blockade, resulted in enhanced tumor control and extended survival. The study suggests further investigation into the potential of statins to enhance responses to PD-1 checkpoint blockade and other immunotherapies in HNSCC [68]. A meta-analysis of 175,000 participants indicated that a 5-year duration of statin therapy had no impact on the occurrence of any type of cancer [69]. The exact mechanisms by which statins influence cancer development and progression are still under investigation.

Table 1 highlights the major Randomized Controlled Trials (RCTs) conducted to study the effects of statins.

## 3. Adverse Effects of Statin Therapy

**Figure 6:**
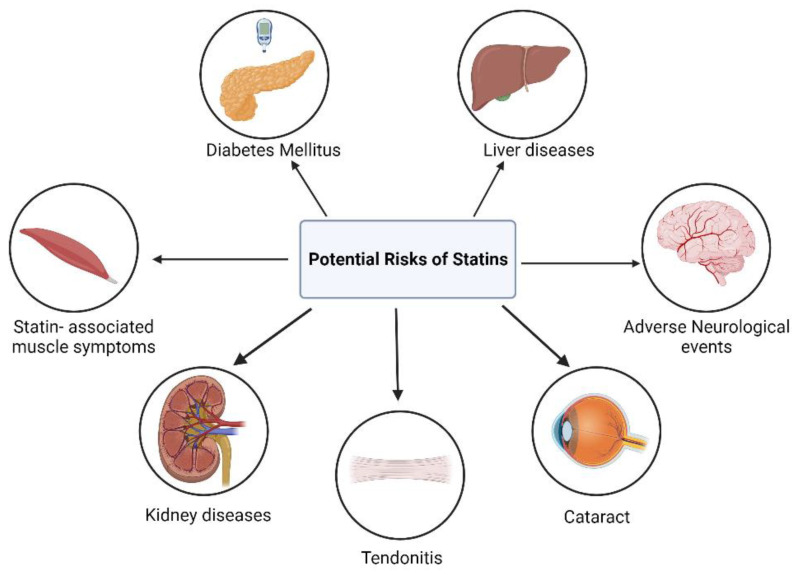
Potential Risks associated with the use of Statins. Created with Biorender.com. Accessed on 6 October 2023.

Statin therapy has frequently been associated with several unintended adverse effects, which further contribute to the concept of statin pleiotropy (Figure 6).

### 3.1. Myopathy and Rhabdomyolysis

The most common side effect associated with statin use is muscle-related symptoms. Myopathy is typically characterized by muscle pain, tenderness, or weakness, accompanied by a significant increase in blood creatine kinase (CK) levels, which frequently exceed ten times the upper limit of normal (ULN) in laboratory tests. Creatine kinase is an enzyme released when muscle cells are damaged. Rhabdomyolysis is a severe form of myopathy where CK levels exceed 40 times the ULN. This condition involves the breakdown of muscle tissue, releasing myoglobin into the bloodstream. This can potentially result in sudden kidney failure or impaired renal function. This condition is characterized by significantly higher elevations in creatine kinase levels [74,75]. Preclinical research suggests that statins can reduce mitochondrial activity, lower energy production, and impact muscle protein breakdown, potentially linking statin use to muscle-related symptoms [76]. Clinical and scientific studies have been used to investigate the mechanisms underlying muscular side effects associated with statin therapy [77]. Muscle biopsies of statin-treated patients with normal creatine kinase (CK) levels revealed mitochondrial dysfunction, lipid accumulation, and structural changes. Basic studies explored alterations in muscle chloride channels, atrogin-1 gene induction, and changes in calcium signaling. The genetic variant Asp247Gly in the LILRB5 gene was linked to an increased risk of statin intolerance [78]. Atrogin-1 induction by statins causing muscle damage may be counteracted by overexpression of PPAR-γ coactivator-1alpha (PGC-1 α), potentially mitigated by metformin. The study suggests exploring metformin’s role in preventing adverse effects of statins and highlights the intricate molecular mechanisms involved in statin-induced myopathy [79].

While infrequent, statin-induced myopathy, characterized by a substantial increase in serum creatine kinase (CK) levels, is a significant adverse outcome associated with statin use. Observational and randomized studies suggest that between 1 in 1000 and 1 in 10,000 patients receiving standard statin doses may experience these side effects each year. In the case of rhabdomyolysis, which affects 2–3 cases per 100,000 patients treated annually, this occurrence is notably less common [80]. Observational studies suggest that the significant prevalence of SAMS may potentially diminish the cardiovascular benefits derived from statin use [81,82]. Consequently, conducting further research to understand the pathophysiology of SAMS is imperative.

### 3.2. Diabetes Mellitus

Data from RCTs show an increased incidence of diabetes mellitus during statin therapy is due to the patients who are already at a higher risk of diabetes progressing to diabetes earlier than they would have otherwise [83,84]. Patients develop chronic insulin resistance and experience a progressive loss of beta-cell function over an extended period of time, leading to the development of type 2 diabetes mellitus [85]. The JUPITER trial observed elevated levels of glycated hemoglobin in individuals taking rosuvastatin, along with a slight increase in the occurrence of diabetes mellitus (3.0% vs. 2.4%, *p* = 0.01) compared with those on placebo [58,86]. Additionally, there was a slight increase in the number of newly identified diabetes cases. This surplus of diabetes diagnoses primarily occurred shortly after starting statin therapy among individuals with significant diabetes risk factors, such as a higher body mass index, elevated HbA1c levels, or impaired fasting glucose. Notably, this increase did not intensify as treatment continued [58,87]. Although statin therapy does elevate the risk of developing diabetes mellitus, the exact mechanism behind this remains uncertain. Nevertheless, the use of statins in individuals at high risk of CVDs should not be discouraged.

### 3.3. Liver Diseases

The research on the effects of statins on liver disease has demonstrated both potential benefits and concerns. On the positive side, statins have been associated with improvements in liver enzyme levels and a potential reduction in the progression of non-alcoholic fatty liver disease (NAFLD) [88]. On the contrary, initial clinical trials of statins observed increased aminotransferase levels in around 2% of patients. A common side effect, often resolving when the dosage is reduced, is the asymptomatic elevation of hepatic enzyme activity [89]. Despite their widespread use worldwide, acute liver failure has been rare. However, Statin-induced drug-induced liver injury (DILI) causing acute liver failure (ALF) remains a concern [90]. Numerous retrospective investigations and randomized controlled trials have indicated that patients with preexisting liver conditions can use statins without endangering their health. However, it is advisable to avoid their use in cases of liver failure, acute liver injury, or decompensated cirrhosis [91].

### 3.4. Adverse Neurological Events

Neurological conditions associated with statin use include hemorrhagic stroke, cognitive decline, peripheral neuropathy, depression, memory issues, aggression, and personality changes [92]. The impact of statins on intracerebral hemorrhages (ICH) has been debated, with some studies suggesting a potential risk increase [31], while recent comprehensive research and meta-analyses did not find a clear association between statin use and ICH [93,94,95]. 

The impact of statin medication on memory loss has been investigated with varying findings. Some studies suggest that statin use could lower the risk of cognitive decline or dementia, including Alzheimer’s disease. These potential neuroprotective effects are hypothesized to be related to statins’ anti-inflammatory and antioxidant properties, which may benefit brain health. However, in some studies, no compelling evidence was discovered indicating a connection between statins and Alzheimer’s disease or cognitive function [96,97]. Reduced serotonin activity, linked to low cholesterol levels, may lead to increased impulsivity, aggression, and depression [98]. Further research is needed to determine whether these reported psychological changes can be attributed to low cholesterol levels.

### 3.5. Cataract

Observational data, along with limited preclinical research, suggested a potential link between the use of statins and the development of cataracts. In a clinical investigation, the researchers highlighted that triparanol could trigger cataracts in both white rats and human subjects. As a result of the occurrence of cataracts and other side effects in individuals in various age groups, the therapeutic application of triparanol was discontinued [99]. In a prospective cohort study encompassing more than two million individuals aged 30 to 84 years, it was observed that 20% had either previously used or were currently using statins throughout the study duration. The findings from this study indicated that both men and women using statins were at a higher risk of cataract development [100]. Notably, there was no evidence to suggest a dose-response relationship. Furthermore, when examining the risk over time, it was discovered that the risk of cataract development substantially increased within the first year of initiating statin therapy. This elevated risk persisted throughout the course of treatment and then decreased significantly within the first year after the discontinuation of statin therapy [101]. Based on the findings of a recent meta-analysis of these studies, it can be concluded that there is insufficient compelling evidence to support the idea that statin use significantly increases the occurrence of cataracts. There is ongoing debate about how statin use affects cataract development.

### 3.6. Kidney Diseases

According to a study conducted by the Canadian Network for Observational Drug Effect Studies (CNODES), individuals taking high-potency statins, as opposed to low-potency ones, faced a 34% higher risk of being hospitalized for acute kidney injury (AKI) within 120 days of starting treatment [102]. In the first two years of commencing lower-dose statin medication, approximately 1 in 500 patients needed hospitalization for AKI. For those using more potent statins during the same period, there was a 15% higher relative risk of experiencing renal damage [102]. Nonetheless, in a meta-analysis comprising 57 randomized controlled trials (RCTs) involving nearly 140,000 patients who were prescribed statin medication for a minimum of six months, it was observed that the rate of estimated glomerular filtration rate (eGFR) decline slowed down by 0.41 mL/min per 1.73 m^2^ annually (with a 95% CI ranging from 0.111 to 0.70) [103]. Furthermore, statin therapy led to a decrease in the standardized mean difference in the alteration of albuminuria or proteinuria among approximately 5000 patients in 29 trials that provided such data, with a reduction of 0.65 standard deviations (95% CI −0.94–−0.37) compared with control groups. However, it is important to note that based on the findings from RCTs, the use of statin medication did not show any noticeable impact on the development of end-stage renal disease [26,103]. It is worth mentioning that various observational studies have reported diverse effects of statin use on kidney function. Some studies suggest a decreased risk, while others indicate an increased risk, and some show no significant changes. The effects of statins on renal function remain a topic of controversy with conflicting findings [104]. While statins may have a protective impact on the kidney, additional research is required for a more definitive conclusion.

### 3.7. Tendonitis and Tendon Rupture

According to numerous studies and case reports, statins may increase the risk of tendon rupture [105]. In a case-control study conducted, exposure to statins was compared between 93 cases of tendon rupture and 279 sex- and age-matched controls. There was no significant difference in statin use rates between cases and controls. However, subgroup analysis revealed that statin exposure was a significant risk factor for tendon rupture in women but not in men [106]. Moreover, in a retrospective cohort study involving over 800,000 individuals aged 64 or older, each with at least a year of continuous enrollment, statin users were paired with two comparable controls in terms of age and gender after initiation of statin therapy. The study compared baseline characteristics and risk factors for tendon rupture between the treatment and control groups. The incidence of tendon rupture did not significantly differ between statin users and non-users. Even after adjusting for age and gender, the results remained consistent, with 69,498 controls matched to 34,749 individuals on statin therapy [107]. Based on this study and numerous others, there is no clear established link between the usage of statins and the occurrence of tendon rupture [108]. 

The 2015 review by Šimić et al. delves into the adverse effects of statins, addressing concerns about their safety amidst widespread use [109]. While recognizing their generally good safety profile, there is a need for critical examination. Key findings, including the rarity of myopathy and rhabdomyolysis, occasional reversible liver enzyme elevation, and potential hepatic steatosis improvement, are highlighted. The nuanced relationship between statins and type 2 diabetes is discussed, emphasizing cardiovascular benefits outweighing diabetes risks. This review explores adverse renal effects, dispels concerns about increased cancer risk, and suggests potential cancer-related benefits. Addressing initial worries about cognitive dysfunction, recent data hint at a positive impact on dementia prevention. Overall, this review systematically clarifies statins’ adverse effects, contributing to understanding their safety profile and underscoring their importance in managing cardiovascular health.

## 4. Conclusions and Future Perspectives

Data reported in the present review suggests that statin therapy is highly effective at reducing the risk of cardiovascular events in both primary and secondary prevention. Both basic and clinical research conducted to date have indicated the presence of distinct secondary effects of statins that their primary mode of action cannot fully explain. While preclinical studies have shown positive effects on endothelial function, platelets, vascular smooth muscle, and inflammation, these benefits have not consistently translated into significant results in large-scale randomized controlled trials. However, the use of statins has been associated with various potential side effects based on clinical investigations and research. These include muscle damage, liver issues, an increased risk of diabetes, cognitive impairment, and hemorrhagic stroke. One serious side effect is myopathy, characterized by unexplained muscle soreness or weakness. The occurrence of myopathy, including rhabdomyolysis, is extremely rare, happening in less than 0.1% of patients when prescribed at the highest doses. SAMS are frequently reported, but data from meta-analyses of RCTs indicate that the prevalence is exceedingly low. The study also suggests that regular statin use slightly elevates the risk of developing diabetes after an initial diagnosis. Severe liver toxicity from statins is exceptionally rare. Statins may potentially increase the risk of hemorrhagic stroke in patients with a history of cerebrovascular disease. Several other side effects, such as peripheral neuropathy, cognitive issues, tendon rupture, cataracts, and AKI, have limited evidence linking them to statin use.

Healthcare professionals should be vigilant about the potential risks and monitor their patients for any signs of adverse effects. It is essential to recognize that the benefits of statin medication far outweigh any potential risks or safety concerns. However, when prescribing these medications, patient-specific conditions should be carefully considered. It is essential for individuals to be well-informed about possible side effects and to have open discussions with their healthcare providers regarding any concerns.

In recent years, numerous non-statin options have come to the forefront, offering alternative approaches to managing lipid levels and reducing cardiovascular risk. These therapeutic options encompass PCSK9 inhibitors, bile acid sequestrants, ezetimibe, and emerging medications such as bempedoic acid [110,111,112]. Clinical trials have demonstrated their efficacy in reducing LDL cholesterol levels, especially in patients with conditions such as familial hypercholesterolemia or those who are unable to tolerate statins. PCSK9 inhibitors have shown remarkable potential in substantially lowering LDL cholesterol [111]. As research progresses and more affordable options become available, non-statin lipid-lowering therapies are poised to play an increasingly significant role in comprehensive cardiovascular risk management, providing additional choices for both patients and healthcare providers. In addition, the emergence of forthcoming lipid-lowering medications under development offers an opportunity to gain additional understanding regarding whether these non-LDL-cholesterol-lowering characteristics are exclusive to statin therapy.

Insufficient conclusive evidence exists for various attributed adverse effects of statins, and any proven or unproven negative impacts are significantly outweighed by the beneficial effects of statins in preventing cardiovascular disease. 

## Figures and Tables

**Table 1:** Major RCTs in the field.

Study Title	Participants	Intervention	Duration	Comparison	Main Findings
1. HPS (Heart Protection Study) [70]	20,536 patients (High-risk individuals)	Simvastatin 40 mg/d	5.3 years	Placebo	This randomized controlled trial assessed the impact on 11-year mortality and morbidity. Statin use reduces major vascular events.
2. ASCOT-LLA (Anglo-Scandinavian Cardiac Outcomes TrialLipid-Lowering Arm) [71]	10,305 patients (Hypertensive patients)	Atorvastatin 10 mg/d	Mean 3.3 years	Placebo	Atorvastatin reduces cardiovascular events in hypertensive patients.
3. JUPITER (Justification for the Use of Statins in Prevention: An Intervention Trial Evaluating Rosuvastatin) [20]	17,802 participants (High-risk individuals)	Rosuvastatin 20 mg/d	Average 2 years	Placebo	Rosuvastatin reduces cardiovascular events in individuals with elevated hs-CRP without a systematic rise in reported adverse events.
4. PROVE-IT TIMI 22 (Pravastatin or Atorvastatin Evaluation and Infection Therapy-Thrombolysis in Myocardial Infarction 22) [72]	4162 patients (post-ACS patients)	Pravastatin 40 mg/d vs. Atorvastatin 80 mg/d	Median 2 years	Intensive vs. moderate statin therapy	Intensive statin therapy (Atorvastatin) reduces cardiovascular events more than moderate therapy (Pravastatin).
5. TNT (Treating to New Targets) [73]	10,001 patients (stable coronary heart disease)	Atorvastatin 10 mg/d vs. 80 mg/d	Median 4.9 years	Standard vs. high-dose statin therapy	High-dose Atorvastatin is more effective in reducing cardiovascular events.
